# When Visual Cues Do Not Help the Beat: Evidence for a Detrimental Effect of Moving Point-Light Figures on Rhythmic Priming

**DOI:** 10.3389/fpsyg.2022.807987

**Published:** 2022-02-04

**Authors:** Anna Fiveash, Birgitta Burger, Laure-Hélène Canette, Nathalie Bedoin, Barbara Tillmann

**Affiliations:** ^1^Lyon Neuroscience Research Center, CNRS, UMR 5292, INSERM, U1028, Lyon, France; ^2^University of Lyon 1, Lyon, France; ^3^Institute for Systematic Musicology, University of Hamburg, Hamburg, Germany; ^4^University of Burgundy, F-21000, LEAD-CNRS UMR 5022, Dijon, France; ^5^University of Lyon 2, Lyon, France

**Keywords:** rhythm, entrainment, rhythmic priming, audio-visual, auditory-motor, syntax, grammar, language

## Abstract

Rhythm perception involves strong auditory-motor connections that can be enhanced with movement. However, it is unclear whether just seeing someone moving to a rhythm can enhance auditory-motor coupling, resulting in stronger entrainment. Rhythmic priming studies show that presenting regular rhythms before naturally spoken sentences can enhance grammaticality judgments compared to irregular rhythms or other baseline conditions. The current study investigated whether introducing a point-light figure moving in time with regular rhythms could enhance the rhythmic priming effect. Three experiments revealed that the addition of a visual cue did not benefit rhythmic priming in comparison to auditory conditions with a static image. In Experiment 1 (27 7–8-year-old children), grammaticality judgments were poorer after audio-visual regular rhythms (with a bouncing point-light figure) compared to auditory-only regular rhythms. In Experiments 2 (31 adults) and 3 (31 different adults), there was no difference in grammaticality judgments after audio-visual regular rhythms compared to auditory-only irregular rhythms for either a bouncing point-light figure (Experiment 2) or a swaying point-light figure (Experiment 3). Comparison of the observed performance with previous data suggested that the audio-visual component removed the regular prime benefit. These findings suggest that the visual cues used in this study do not enhance rhythmic priming and could hinder the effect by potentially creating a dual-task situation. In addition, individual differences in sensory-motor and social scales of music reward influenced the effect of the visual cue. Implications for future audio-visual experiments aiming to enhance beat processing, and the importance of individual differences will be discussed.

## Introduction

The majority of research investigating rhythm processing (both perception and production) occurs in the auditory modality, as rhythm and beat processing are typically more precise and more frequent in the auditory domain (Grahn, 2012; Repp and Su, 2013; Comstock et al., 2018). However, attempts have been made to investigate whether regular visual information can effectively convey a rhythmic beat, both independently and in combination with auditory conditions. Research has shown that synchronization to visual stimuli with a visible movement trajectory (e.g., a tapping hand, a bouncing ball) is enhanced compared to discrete visual stimuli (e.g., flashing lights), and can reach the performance level of synchronization to auditory stimuli (Hove and Keller, 2010; Hove et al., 2013; Gan et al., 2015). Importantly, movement of the participant appears to be necessary to elicit equal performance between moving visual cues and auditory cues. Across different studies, bouncing balls (i.e., a moving visual stimulus) were superior to visual flashes (i.e., a static stimulus) and elicited similar performance to auditory stimuli (tones/beeps) in beat-based synchronization tasks, but not in beat-based perception tasks (Silva and Castro, 2016; Torres et al., 2019; Gu et al., 2020). These studies suggest that moving visual cues can match auditory stimuli only when the participant is engaged in a motor task (e.g., tapping synchronization), likely activating sensorimotor (visual-motor) networks (Gu et al., 2020).

### Biological Motion and Beat Perception

As apparent movement (i.e., a bouncing ball) appears important to show a visual cue benefit for synchronization tasks, it is also possible that visual stimuli reflecting *body movement* (i.e., biological motion) might enhance the involvement of auditory-motor coupling in the brain, even without additional participant movement. The auditory-motor pathway (i.e., the connection between auditory and motor cortices) appears integral to beat perception and temporal prediction (Patel and Iversen, 2014; Morillon et al., 2015; Proksch et al., 2020; Cannon and Patel, 2021), as just *listening* to rhythms activates motor areas in the brain (Grahn and Brett, 2007; Chen et al., 2008; Gordon et al., 2018), and the motor system is actively involved in music perception (Maes et al., 2014). Studies have shown that adding participant movement to auditory stimuli can influence auditory perception (Phillips-Silver and Trainor, 2007; Brown and Palmer, 2012; Chemin et al., 2014; Mathias et al., 2015, 2016; Schmidt-Kassow et al., 2019), and actively engaging the motor system in beat-based processing, for example by tapping or moving along to a beat, enhances rhythm perception (Manning and Schutz, 2013). Further, participant pairs facing each other while synchronizing (flexing and extending knees) to an auditory metronome were more in-synch compared to when they were facing apart, a benefit attributed to the continuous visual cue of their partner (e.g., Miyata et al., 2017). However, limited research has investigated whether the addition of a biological motion visual cue without additional participant movement can enhance the auditory-motor loop and/or enhance perception.

Visual point-light figures have been shown to enhance auditory rhythm perception and synchronization. Su (2014b) used point-light figures (very similar to the ones used in our current study) that bounced in time with auditorily presented rhythms. Experiment 1 (*n* = 14) consisted of a same-different judgment task (i.e., beat-based perception) and Experiment 2 (*n* = 11 plus author) consisted of a synchronization task. Short, metrically simple rhythms [with five to seven intervals taken from Grahn and Brett (2007) and Grahn (2012)] were used. Rhythms were either presented alone (auditory condition) or with the point-light figure (audio-visual condition). In the same-different task, participants were presented with the same rhythm three times (with or without a point-light figure), and the third time they judged whether the third rhythm was the same or different. Participants performed better at the same-different task in the audio-visual condition compared to the auditory condition, suggesting a benefit of the point-light figure on rhythm perception. Similarly, in the synchronization task, participants were less variable in tapping to isochronous rhythms in the audio-visual condition compared to the auditory condition. For both experiments, auditory distractor sequences of different tempi were included that participants were told to ignore, but which made the task progressively more difficult. Interestingly, in both experiments, as performance in the auditory condition decreased, the effect of the visual stimulus increased, showing that the visual cue was only beneficial as the auditory rhythms became more difficult. In other words, the visual cue may have only been used when the beat was not easily extracted from the auditory stimulus.

However, other studies have not shown beneficial effects of visual biological motion (i.e., human movement or point-light figures portraying humans) on beat-based perception. Using the same visual point-light figures as Su (2014b) and short, weakly metrical sequences from Grahn and Brett (2007), Su (2014a) had participants perform a reproduction task (Experiment 1, *n* = 12 plus author) or a same-different task (Experiment 2, *n* = 19). In these experiments, the auditory (auditory rhythm), visual (point-light figure), or audio-visual (auditory and point-light figure) beat was provided for two beats before being presented simultaneously with the starting beat of the weakly metrical rhythms. Across both experiments, there was no benefit of the audio-visual condition compared to the auditory condition, showing that the visual cue did not help reproduction or differentiation of the weakly metrical sequences. Su (2014a) suggested that presenting the stimuli in multiple streams might have led to a high working memory load, which could have removed any effects of beat induction in the weakly metric rhythms. Additional studies in adults (Phillips-Silver and Trainor, 2007, 2008) and infants (Phillips-Silver and Trainor, 2005) showed that when participants bounced (or were bounced) with an ambiguous rhythmic sequence at either a duple (i.e., bounce every second beat) or triple (i.e., bounce every third beat) meter, their recognition of and liking for the unambiguous sequences of the same meter increased. However, passively watching an experimenter bouncing did not influence subsequent recognition (adults) or liking for (infants) unambiguous sequences in the meter that was bounced to, suggesting that beat-based perception alone was not affected by the visual cue. The literature is therefore conflicting as to whether adding a visual point-light animation without additional movement by the observer can aid auditory beat-based processing.

The current study investigated whether the addition of a moving point-light figure (i.e., with biological motion) could enhance beat-based perception within a rhythmic priming paradigm. Point-light animations are an interesting stimulus to study audio-visual movement as they have the potential to enhance the activation of auditory-motor connections in the brain. Just watching moving human point-light displays has been shown to activate the premotor cortex (Saygin et al., 2004; Saygin, 2007), and engage the mirror neuron system (Ulloa and Pineda, 2007; see also Copelli et al., 2021). Further, motor system activity is increased when participants are presented with actions that they can perform compared to those they cannot perform (Stevens et al., 2000), likely related to the strong link between action and action perception in the brain (Case et al., 2015). Additionally, human movement is typically perceived as more socially relevant and salient than object movement (i.e., bouncing balls) or non-human movement (i.e., moving horses) (Pyles et al., 2007; Pinto and Shiffrar, 2009). The ability of point-light animations to communicate biological movement is also shown in developmental studies. Children as young as 3-years-old can recognize point-light figures when they are moving but not when static, with ceiling recognition performance already at 5 years of age (Pavlova et al., 2001), and 12-month-old infants spontaneously follow the gaze direction of point-light figures (Yoon and Johnson, 2009). Such results suggest that point-light animations are adept at communicating human movement and social gestures. Taken together, such evidence suggests that watching a human point-light figure moving in a synchronous and physically plausible manner might strengthen the involvement of the auditory-motor connection in the brain during perception, thereby potentially enhancing participants’ beat-based perception (as shown in Su, 2014b). Our aim was to recreate the beneficial conditions in Su (2014b) by using visual point-light animations and strongly metrical sequences to improve beat-based rhythm perception within a rhythmic priming paradigm.

### Rhythmic Priming

A growing body of literature has shown that presenting a regular rhythmic prime before a set of naturally spoken sentences enhances grammaticality judgments for these sentences compared to irregular rhythmic primes or baseline conditions. A rhythmic priming effect has been shown for French speaking children (Przybylski et al., 2013; Bedoin et al., 2016; Fiveash et al., 2020), French speaking adults (Canette et al., 2019, 2020a), English speaking children (Chern et al., 2018) and Hungarian speaking children (Ladányi et al., 2021). This effect appears to be driven by a benefit to syntax processing after regular rhythms rather than a detrimental effect after irregular rhythms, as shown with environmental sounds, contemporary textural music, and silence as baseline comparison conditions (Bedoin et al., 2016; Canette et al., 2020b; Ladányi et al., 2021). Further, the effect appears specific to syntax processing rather than a general arousal effect, as no benefit of the regular rhythms was found for a non-linguistic control task (Chern et al., 2018; Ladányi et al., 2021) or a semantic evocation task (Canette et al., 2020b). These studies used longer rhythmic primes (17–32 s) compared to previous cueing studies (e.g., Cason and Schön, 2012; Cason et al., 2015; Falk and Dalla Bella, 2016) with the aim to globally entrain endogenous neural oscillations to the external regular rhythms.

Within the framework of dynamic attending theory (Large and Jones, 1999; Jones, 2018), the entrained neural oscillations should persist once the rhythm stops, enhancing the processing of the subsequently presented naturally spoken sentences. The strength of the entrained oscillations should determine how long the oscillation persists, with stronger driven oscillations persisting for a longer period of time after the end of the input, and less likely to be captured by new rhythms or events (Jones, 2018; Fiveash et al., 2020). Therefore, enhancing the entrainment to the regular rhythmic primes should also enhance the rhythmic priming effect, with stronger entrainment resulting in prolonged effects of the regular rhythmic primes on subsequent sentence processing. Previous research has shown that adding a motor component (tapping along or rhythmic training) to rhythmic cueing studies (i.e., with a one-to-one match of the cue and target as in Cason and Schön, 2012) can enhance the effect of the rhythmic cue on subsequent speech perception (Cason et al., 2015; Falk and Dalla Bella, 2016; Falk et al., 2017). However, to our knowledge, no studies have yet investigated whether rhythmic cueing or rhythmic priming can be enhanced with the addition of a visual cue in the absence of participant movement. Visual cues depicting human movement could be particularly valuable as they might lead to enhanced involvement of auditory-motor coupling and its related contribution to beat and meter processing.

### Current Study

The current set of experiments aimed to investigate whether adding a visual point-light figure moving in time to regular rhythms could enhance the rhythmic priming effect by enhancing entrainment to the regular rhythms. Based on the links between point-light figures and motor activation in the brain (Saygin et al., 2004; Saygin, 2007), and the capacity of the point-light figures for enhancing beat-based processing for strongly metrical stimuli (Su, 2014b), we decided to create point-light figures similar to those used in Su (2014a,b) as our visual stimulus. However, as reviewed above, the evidence supporting beneficial effects of visual cues without additional movement from the participant is limited. Therefore, there are three possible outcomes for the following experiments comparing regular audio-visual primes to either regular auditory primes (Experiment1) or irregular auditory primes (Experiments 2 and 3). The first possible result pattern would reflect a *beneficial* effect of the visual cue on beat-based perception. In this case, regular audio-visual primes should result in improved grammaticality judgments compared to both regular auditory primes and irregular auditory primes. The second possible result pattern would reflect *no effect* of the visual cue on beat-based perception. In this case, grammaticality judgments should be equal after regular audio-visual primes and regular auditory primes, and still result in enhanced performance compared to irregular primes (i.e., the rhythmic priming effect). The third possible result pattern would reflect a *detrimental* effect of the visual cue on beat-based perception (i.e., linked to an additional information cost if not integrated with the auditory information). In this case, regular audio-visual primes should result in poorer grammaticality judgments compared to regular auditory primes, and the same or lower performance compared to irregular primes.

Experiment 1 was conducted with children and directly investigated whether adding a visual point-light figure *bouncing* in time to regular rhythms would enhance grammaticality judgments compared to the same regular rhythms presented only in the auditory modality. This experiment was therefore a direct test of whether the visual cue enhances rhythmic priming compared to the same cue without a visual component. To preface the results, Experiment 1 showed that the regular audio-visual rhythms resulted in *poorer* performance on the grammaticality judgment task compared to the regular auditory rhythms (supporting the detrimental effect hypothesis). We hypothesized that children were more likely to be distracted by the addition of a visual point-light figure and may have difficulties integrating the two types of information. They may have thus processed the stimuli as a dual-task rather than an integrated percept. Therefore, we ran Experiments 2 and 3 with adults.

Experiments 2 and 3 built more directly on the priming conditions initially used in previous studies (e.g., Przybylski et al., 2013; Chern et al., 2018; Fiveash et al., 2020), and thus consisted of regular audio-visual primes and irregular auditory primes. Irregular auditory primes were introduced to have a stronger contrast with the regular audio-visual primes and to investigate whether the regular audio-visual primes still elicited the rhythmic priming effect. To further investigate the role of the visual cue and in particular its benefit over a purely auditory regular rhythm, we compared these data to previous adult data using regular and irregular auditory rhythms without visual cues (Canette et al., 2019). The visual cue in Experiment 2 was the same bouncing point-light figure as in Experiment 1. To investigate whether the specific movement of the visual cue was important, the point-light figure in Experiment 3 was changed to a swaying figure which had more precise alignment of the hip movement to the beat onsets. None of the experiments showed a benefit of the regular audio-visual prime on grammaticality judgments, supporting previous research suggesting no benefit of purely visual cues on beat-based perception. On the contrary, the results indicated that the addition of the visual information reduced the typical rhythmic priming benefit, reflecting a detrimental effect of the visual cues. However, individual differences appeared to influence the impact of the visual cue and will be outlined below (including perspectives for conditions leading to potential benefits).

## General Method

### Design

**FIGURE 1:**
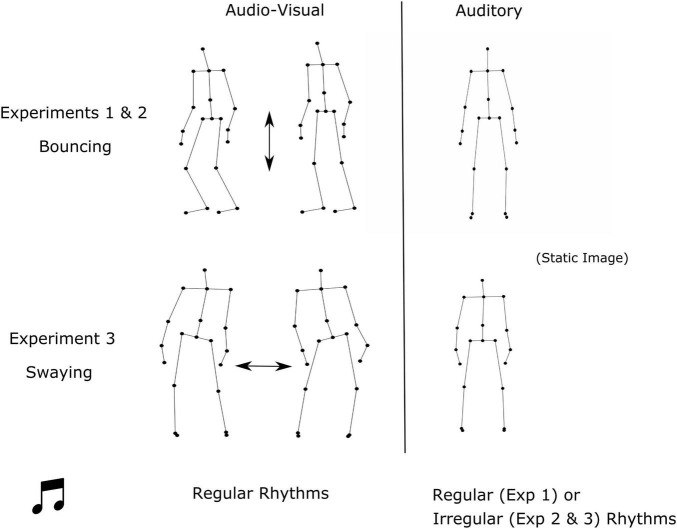
Diagram showing the audio-visual (AV) and auditory conditions for the rhythmic priming experiments. The AV condition in Experiments 1 and 2 consisted of a bouncing figure that bounced up and down in time with the music. In Experiment 3 the AV condition consisted of a swaying figure that swayed side to side. The AV condition consisted of regular rhythms for all experiments, whereas the auditory condition consisted of regular rhythms in Experiment 1 and irregular rhythms in Experiments 2 and 3.

All experiments were 2 (condition: audio-visual, auditory) by 2 (sentences: grammatical, ungrammatical) within-subject designs. Across all experiments, auditory rhythms were paired with a static visual image of the point-light figure so that they also contained visual information. In Experiment 1, children (aged 7–9 years) listened to regular rhythms presented simultaneously with a point-light figure that *bounced* in time with the underlying beat of the music, referred to as audio-visual rhythms (RegAV), or regular rhythms presented simultaneously with a static visual image, referred to as auditory rhythms (RegA). In Experiment 2, adults were presented with RegAV rhythms with the same bouncing point-light figure as in Experiment 1, or *irregular* auditory rhythms (IrregA) presented with the same static image. In Experiment 3, adults were presented with RegAV rhythms with a *swaying* point-light figure, or IrregA rhythms with the same static image. See Figure 1.

Each experimental block consisted of one rhythm (AV or A) followed by six sentences. Experiment 1 contained 16 blocks of one rhythm followed by six sentences, and Experiments 2 and 3 consisted of eight blocks of one rhythm followed by six sentences. Starting condition (AV or A) was counterbalanced across participants, and there were four blocks of the same presentation type in a row aiming to enhance the effect of condition. Two different sentence lists (1, 2) were counterbalanced across participants, and counterbalancing was designed in a way that four different sets of stimulus presentation were possible across participants: list 1 with AV first; list 2 with AV first; list 1 with A first; list 2 with A first. Each block of six sentences contained three grammatical and three ungrammatical sentences. Within these constraints, all music and speech stimuli were fully randomized.

### Stimuli

#### Rhythms

The regular and irregular experimental rhythms were the same as those used in Fiveash et al. (2020), and were approximately 32 s long. Three additional rhythms (created by the same composer) were used for the training phase to familiarize participants with the bouncing/swaying figures. All rhythms were created with musical instrument digital interface virtual studio technology (MIDI VST) and contained various percussive and electronic sounds (i.e., bass drum, snare drum, tom-tom, cymbal). Regular rhythms had a 4/4 meter, and a tempo of 120 beats per minute (bpm). Irregular rhythms were created from the regular rhythms by re-arranging the events in time, so that the sequences were highly irregular, with no underlying meter or pulse. See Supplementary Material for examples of the regular and irregular rhythms, as well as the Supplemental Information in Canette et al. (2019) for more rhythmic examples.

#### Point-Light Animations

For the audio-visual condition (RegAV), point-light animations were created using an infrared-based motion capture system (Qualisys Oqus 5+, 8 cameras, Qualisys Track Manager 14 software). To create the figures, a musically trained female was equipped with 28 reflective markers and bounced up and down (for bouncing stimuli, Experiments 1 and 2) or swayed side to side (for swaying stimuli, Experiment 3), with the knee flexion (bouncing) or hip extension (swaying) aligning with each beat. Movement was recorded at a frame rate of 120 frames per second, labeled, and exported to Matlab 2017b. Subsequently, markers were reduced to 20 joints (to avoid redundancies and create a clearer image, for more information on this procedure, see Burger et al., 2013), and rendered as .mov videos on a white background with black connectors between the points. To enhance the bouncing motion and to make knee movement clear, the point-light figure was rotated 45° to the left (when facing the figure) in the bouncing AV condition. For swaying, the point-light figure was facing forward, allowing for better viewing of the movement. The audio was added using QuickTime Player, v.10, ensuring correct (i.e., natural and aligned) synchronization between rhythm and movement. See Supplementary Material for example animations. For the auditory conditions, a forward-facing static image of the same point-light figure was presented on the screen to control for effects of visual information between conditions. In a training phase before the experimental phase, participants were presented with in-synch and out-of-synch moving figures. To create the out-of-synch videos, the point-light figure was sped up to bounce at 160 bpm, and so looked particularly out-of-synch with the rhythm at 120 bpm (this was also confirmed in the pilot experiment presented below).

#### Sentences

Different sentences were used for the child experiment (Experiment 1) and the adult experiments (Experiments 2 and 3) based on different required difficulty levels. The child sentences consisted of two lists of 96 French sentences spoken naturally by a native French speaker and used in Fiveash et al. (2020). Each list contained 48 grammatical and 48 ungrammatical sentences that were matched on lexical properties, including number of words, number of syllables, and lexical frequency. Sentences that were grammatical in List 1 were ungrammatical in List 2, to ensure no effect of individual sentences. There were eight types of grammatical error: number (No), person (Pe), gender (Ge), tense (Te), auxiliary (Au), morphology (MS), position (Po), and past participle (PP). Eight sentences each were composed of the four main error types (No, Pe, Ge, and Te), and four sentences each were composed of the secondary error types (Au, MS, Po, and PP). Within each block of six sentences, there were always three grammatical and three ungrammatical sentences. The three ungrammatical sentences always included two different main error types, and one secondary error type. Further details and a list of all sentences can be found in Fiveash et al. (2020), and example sentences are presented in Supplementary Table 1.

The adult sentences (Experiments 2 and 3) were those used in Canette et al. (2019). The same creation of lists was conducted as in Experiment 1: the incorrect sentences were derived from correct sentences and separated into different lists, so participants did not hear the same sentence in both its correct and incorrect form. Sentences in each list were matched for number of words, number of syllables, and for lexical frequency. In this stimulus set, each list contained 48 sentences (24 grammatical, 24 ungrammatical). Grammatical errors were morpho-syntactic (including tense, preposition, and person agreement errors) and subtle to increase difficulty for the adults. More details and the full stimulus set can be found in Canette et al. (2019); example sentences are presented in Supplementary Table 1.

Experiment 1 had more sentences (and therefore experimental blocks) than Experiments 2 and 3 because it was possible to include more diverse syntactic errors for children, as they do not perform at ceiling level on grammaticality tasks. For adults, creating syntactic errors that are subtle and do not result in ceiling or floor effects is challenging, limiting the number of sentences available. We here used available stimuli from Canette et al. (2019) that had shown a rhythmic priming effect in adults previously.

### Training Phase

To enhance the auditory-motor link, a training phase was introduced to engage participants with the experiment and to familiarize them with the concept of synchronization. In the training phase, participants were told that they would see two different dancers. The first dancer was introduced as a *good* dancer. The good dancer was always in-synch with the music, and consisted of black dots on a white background, as in the main experiment. Participants were told that they could move along with the dancer if they wanted to. The second dancer was introduced as a *bad* dancer who would try to imitate the good dancer. The bad dancer was presented with white dots on a black background to emphasize the difference from the good dancer. Participants saw videos of both dancers. It was explained that participants would see the good dancer followed by the bad dancer, and they had to judge whether the bad dancer did a good job of imitating the good dancer or not. There were four trials, and after each trial, participants verbally indicated to the experimenter whether the bad dancer imitated the good dancer well or not. Half of the time the bad dancer was out-of-synch with the good dancer, and half of the time the bad dancer was in-synch with the good dancer. If the participant indicated the wrong answer, the experimenter explained why the bad dancer was doing a good or bad job of copying the good dancer. All training videos were 8 s long, corresponding to one cycle of the experimental rhythms.

### Individual Differences Tests

To investigate whether individual differences were related to performance after audio-visual or auditory primes, we measured reading age of children (RA), and administered the Barcelona Musical Reward Questionnaire (BMRQ; Mas-Herrero et al., 2013) to adults. Children completed a French age-normed reading measure, the Test de l’Alouette (Lefavrais, 1967), also used in Fiveash et al. (2020). The Test de l’Alouette is a pure measure of reading age (RA), as semantic prediction of the text is largely impossible. Each child had 3 min to read this text out loud. Their score was based on their reading speed (i.e., how much of the text they were able to read) and number of mistakes made, which were then compared to the normed values to calculate RA. Their chronological age (CA) was also recorded. RA was measured for children as previous research has shown connections between RA and the effects of regular rhythmic primes (Fiveash et al., 2020).

Adults completed the French translation of the BMRQ (Saliba et al., 2016), which contains 20 questions corresponding to the sub-scales: musical seeking, emotion evocation, mood regulation, social reward, and sensory-motor. Normed values were calculated at http://brainvitge.org/z_oldsite/bmrq.php. RA or equivalent baseline grammar tests were not implemented for adults, as the population was largely young University students without speech or language disorders who were expected to be relatively homogenous in their reading and grammar level. Music reward was not measured for children as the questions and norms were directed toward an adult audience with more experience in music listening. Children were informally asked whether they had music lessons, but considering the young age range, the small number of years (or months) of music training reported by some children was not analyzed. Both RA (measured in children) and musical reward (measured in adults) were expected to relate to the rhythmic priming effect. More specifically, children with higher RAs were expected to show a rhythmic prime benefit (Fiveash et al., 2020), and perhaps be more positively affected by the audio-visual primes, and adults with higher musical reward (particularly in relation to the sensory-motor sub-scale) were expected to perform better after the audio-visual regular primes compared to the auditory irregular primes, as they were more likely to be engaged with the rhythmic stimuli.

### Procedure

The procedure was similar for all participants, but was adapted depending on age. Children were tested in a quiet room with an experimenter who sat with the child throughout the experiment to ensure adherence to the task and to launch each trial. Adults were tested in a sound-proof booth and progressed through the experiment by themselves. Both children and adults completed the training phase. At the end of the training, the experimenter explained that in the experiment only the good dancer would be dancing, and that sometimes the dancer would be “in form” and dancing, but at other times the dancer would be tired from all the dancing and needed to take a rest. Participants were told that they could move too when the dancer was dancing, but that when the dancer was resting, they should stop moving and listen carefully to the music. They were also told that after the music stopped, they would hear several sentences. For children, it was described that the sentences would either be spoken by a dragon who was always right (correct dragon), or a dragon who was always wrong (confused dragon). Pictures of both dragons were shown next to each other on the screen, and participants heard an example sentence. It was emphasized that the errors would be French errors, not errors of content (e.g., if the dragon said it was snowing outside, but it was not, this would not be an error). For adults, it was described that the sentences would be either grammatically correct or incorrect. After ensuring the participant understood the task, the experimental phase started. For each block, a rhythm (with either a concurrent bouncing/swaying point-light figure or a static point-light figure) was played for 32 s, followed by six sentences presented with the pictures of the dragons (or the words correct/incorrect for adults) on the screen. At the end of each sentence, children indicated whether the clever dragon or the confused dragon had spoken the sentence. Adults indicated whether the sentence was grammatically correct or incorrect. Participants could indicate that there was an error before the end of the sentence. In this case, the dragons (or words correct/incorrect) disappeared from the screen, but the sentence continued until the end. For children, the experimenter pressed a button to continue once the child was ready for the next sentence, and adult participants progressed by pressing the spacebar. After each block, participants were told whether the dancer would dance or whether the dancer needed to take a break in the next block. There was a break after every two blocks. The experiments were run on MacBook Pro laptops, using Matlab (version 2018a) and Psychtoolbox (version 3.0.14). At the end of the experiment, children completed the Test de l’Alouette individually, and adults completed the BMRQ. The full experiment (including the training phase) took approximately 24 min for children and 12 min for adults. Children were encouraged throughout the experiment and given pauses every two blocks to ensure attention.

### Validation of Synchronized Stimuli

To ensure that the experimental videos were perceived as being in-synch with the rhythms, and to ensure that the practice videos were perceived as either in- or out-of-synch as expected, a pilot test was run on eight adult participants with the bouncing stimuli. Participants rated synchronization of the point-light figure with the rhythm on a scale from 1 (very unsynchronized) to 10 (very synchronized). All experimental videos were presented first (randomized for each participant) to ensure they were not influenced by the out-of-synch videos, followed by the practice videos (randomized). Practice videos consisted of three *in-synch* point-light figures with a white background, three *in-synch* point-light figures with a black background, and three *out-of-synch* point-light figures with a black background.

The four experimental rhythms were rated as highly in-synch (*M*_*range*_: 7.25–8.13, *SD*_*range*_: 1.60–2.25). For the practice videos, the three out-of-synch practice videos were rated as highly out-of-synch (*M*_*range*_: 2.38–2.63, *SD*_*range*_: 1.30–1.51). The in-synch practice videos were generally also rated as in-synch, but had more varied ratings (*M*_*range*_: 6.5–8.13, *SD*_*range*_: 1.13–2.27). These data confirmed that the experimental videos were perceived as sufficiently in-synch for the purpose of the experiment, and that the distinction between in-synch and out-of-synch point-light figures was clear for the practice trials.

### Analysis

#### Power Analysis and Sample Size

Sample sizes were determined based on previous rhythmic priming studies which found significant benefits of regular compared to irregular primes. Specifically, previous behavioral sample sizes for typically developing children and adults performing the rhythmic priming task have ranged from *n* = 16 to *n* = 35, with the ability to detect small to medium effect sizes (Canette et al., 2019, *n* = 25, *d* = 0.37; Canette et al., 2020b, *n* = 30, η_*p*_^2^ = 0.30; Chern et al., 2018, *n* = 16, *d* = 0.57; Fiveash et al., 2020, Experiment 2, *n* = 35, *d* = 0.33; Ladányi et al., 2021, *n* = 17, *d* = 0.36). We therefore aimed to follow these conventions and tested approximately 30 participants for each experiment, resulting in 27 children in Experiment 1 (based also on classroom recruitment possibilities), and 31 adults in Experiments 2 and 3.

#### Signal Detection

Detection of grammatical errors was measured by calculating *d* prime (*d*’) from signal detection theory (Stanislaw and Todorov, 1999). D prime provides a measure of sensitivity to the signal which considers both hits (i.e., when there was an error and the participant indicated that there was an error) and false alarms (i.e., when there was no error, but the participant indicated that there was an error). The *d’* value is created by subtracting the *z*-score of the false alarms from the *z*-score of the hits. Extreme hit or false alarm values of one (i.e., 100%) or zero (i.e., 0%) were corrected to 0.99 or 0.01 respectively, as suggested in Stanislaw and Todorov (1999). A measure of response bias (response bias *c*) was also calculated by multiplying the sum of the *z*-scores for hits and false alarms by –0.50. Values above zero suggest a bias to respond grammatical, whereas values below zero suggest a bias to respond ungrammatical.

#### Statistical Analyses

Paired-samples *t*-tests were used for each experiment to judge whether *d*’ values and response bias *c* values differed depending on prior presentation of an audio-visual rhythmic prime (RegAV for all experiments) or an auditory rhythmic prime (RegA for Experiment 1 and IrregA for Experiments 2 and 3). Response bias *c* values were also compared to 0 using one-sample *t*-tests.

The adult data in Experiments 2 and 3 were directly compared to previously published data in Canette et al. (2019), who reported a benefit of the regular compared to irregular rhythmic primes on subsequent grammaticality judgments. Canette et al. (2019) used the same rhythmic primes, the same sentences, and the same design as Experiments 2 and 3. Further, both participant groups consisted of adults of a similar age (in Canette et al., 2019: *n* = 25, *M*_*age*_ = 21.2 years, *SD* = 1.76; range = 19–26). The only differences between the two experiments were that (a) the current experiments included an audio-visual manipulation, and (b) the current experiments presented four of the same prime types in a row (e.g., AAAA BBBB) whereas the previous experiment alternated every two blocks (e.g., AA BB AA BB). To investigate the effect of the visual-cue on performance, independent-samples *t*-tests were conducted to compare (a) performance after RegAV primes in Experiments 2 and 3 to performance after RegA primes in Canette et al. (2019), and (b) performance after IrregA primes in Experiments 2 and 3 compared to the same IrregA primes in Canette et al. (2019)^1^. Respectively, these comparisons allowed us to investigate the effect of adding a visual cue to the regular prime, and to observe whether performance after irregular primes stayed consistent across the experiments. All analyses were run in R studio (R Core Team, 2018).

#### Individual Differences

For Experiment 1, spearman correlations were calculated for RA and CA for each condition (RegAV and RegA). Holm–Bonferroni adjusted *p*-values (*p’*) are presented after correcting for multiple comparisons. These correlations were run separately (i.e., not on the difference score), as we expected a correlation with both RegAV and RegA, based on Fiveash et al. (2020). For Experiments 2 and 3, multiple regressions were run on the difference score of the conditions (RegAV minus IrregA) to investigate whether the sub-scales of the BMRQ were related to the direction of the rhythmic priming effect. The difference score represents the strength of the rhythmic priming effect, and in the current experiments specifically, the strength of the regular audio-visual primes compared to the irregular auditory primes. Values greater than 0 indicate a benefit of the RegAV primes, whereas values less than 0 indicate a benefit of the IrregA primes. We report first the standard regression model (with all sub-scales included: musical seeking, emotion evocation, mood regulation, social reward and sensory-motor), and then the backward stepwise regression model for comparison. Backward stepwise regression (where all sub-scales are included at first, and then those which contribute the least are iteratively removed until there is a model with only significant predictors) was chosen to explore whether the standard model was missing important predictors. We chose backward stepwise regression as it is considered more robust to suppressor effects compared to forward regression (Field et al., 2012).

## Experiment 1

### Participants

Twenty-seven children aged between 7 and 9 years of age (*M* = 95.26 months, *SD* = 3.94 months, range: 90 months–101 months; 7 years, 6 months–8 years, 5 months) from “CE1” grade of a public French school in Lyon, France, participated in this experiment. Informed consent was provided by the parents, and the experiment was run in accordance with the Declaration of Helsinki.

### Results and Discussion

#### D Prime

**FIGURE 2:**
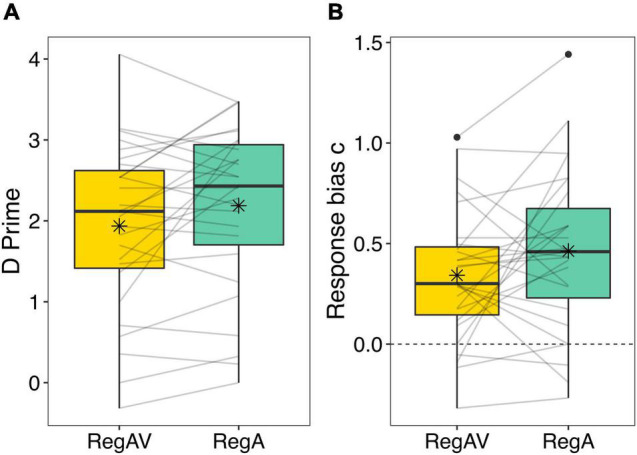
D prime **(A)** and response bias c **(B)** values after regular audio-visual (RegAV) primes and regular auditory (RegA) primes. Individual lines represent individual participant data, and means are represented with a black asterisk. The dotted line for response bias c refers to the point of no bias. Boxplots represent the distribution of data as implemented in ggplot2 in R (R Core Team, 2018), with the black line representing the median, the box representing the interquartile range, the whiskers presenting the spread of data, and extra points representing potential outliers.

The paired-samples *t-*test showed that sensitivity to grammatical errors was significantly worse after a RegAV prime (*M* = 1.94, *SD* = 1.05) compared to a RegA prime (*M* = 2.19, *SD* = 1.02), *t*(26) = 2.09, *p* = 0.047, *d* = 0.40. See Figure 2A. These data show that adding bouncing point-light figures to the regular rhythms significantly reduced performance compared to the regular rhythms alone.

#### Response Bias c

The difference in response bias *c* was not significant depending on condition, *t*(26) = 1.68, *p* = 0.11, as participants were biased to respond grammatical in both the RegAV condition (*M* = 0.34, *SD* = 0.33), *t*(26) = 5.41, *p* < 0.001 and the RegA condition (*M* = 0.46, *SD* = 0.40), *t*(26) = 5.94, *p* < 0.001. See Figure 2B.

#### Reading and Chronological Age

**FIGURE 3:**
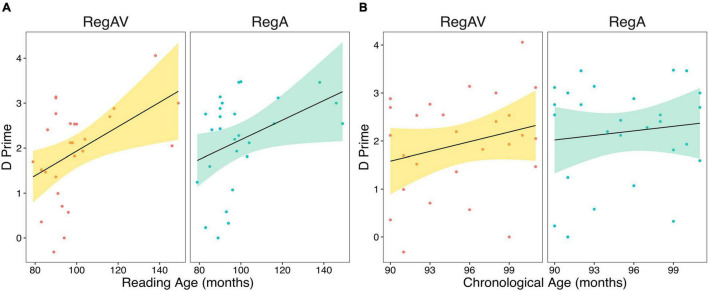
Correlations between d prime performance after regular audio-visual (RegAV) rhythms and regular auditory (RegA) rhythms for **(A)** reading age and **(B)** chronological age. Individual dots represent individual participants. Shaded error bars represent one standard error of the mean, regression line fitted with a linear model in R.

Reading age (in months) was positively correlated with performance after both the RegAV, *r*(25) = 0.46, *p* = 0.015, *p*’ = 0.03 and RegA, *r*(25) = 0.398, *p* = 0.04, *p*’ = 0.079 primes (Figure 3A); however, CA was not [RegAV: *r*(25) = 0.18, *p* = 0.37; RegA: *r*(25) = 0.037, *p* = 0.85], Figure 3B. After correction for multiple comparisons, the correlation between RA and RegA was no longer significant, while the correlation with RegAV remained significant, indicating a slightly stronger correlation^2^ with RA when a visual cue was present.

Experiment 1 showed a detrimental effect of the visual point-light animation on grammaticality judgments, with reduced sensitivity to grammaticality judgments after RegAV primes compared to RegA primes. One possibility to explain this result is that children were disturbed or distracted by the point-light figure, especially with hearing both audio-visual and auditory versions of the same regular rhythms. It is also possible that the visual cue may have become costful and created a dual-task situation, rather than facilitating beat extraction and entrainment. To investigate whether the point-light figure was costful only for children, we conducted a new experiment with adults and compared regular audio-visual and irregular auditory rhythms.

## Experiment 2

### Participants

Thirty-one adults (28 women, three men; *M*_*age*_ = 20.97 years, *SD* = 2.98; range = 19–35) participated in Experiment 2. All were native French speakers and were recruited through the University of Burgundy. On average, participants had 1.69 years (*SD* = 2.73, range = 0–9) of musical experience. Fifteen participants reported some musical experience (seven were still practicing at the time of testing), and 16 participants reported no musical experience. The participants with musical training had an average of 3.5 years (*SD* = 3.04) of courses and playing, ranging from 1 month to 9 years. Eighteen participants reported attending dance classes in the past, and four attended dance classes at the time of testing. One participant reported being dyslexic, and no participants reported a history of neurological, hearing, or vision issues. All participants provided written informed consent, and the study was approved by the French ethics committee (*Comité de Protection des Personnes, Ile de France X, CPP*). Participants were given course credit for their participation.

### Results and Discussion

#### D Prime

**FIGURE 4:**
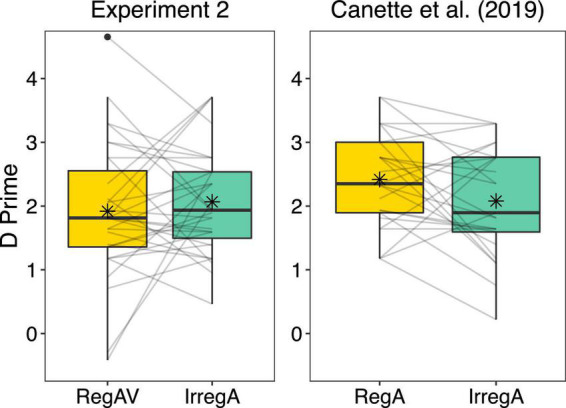
Comparison of the current audio-visual (AV) experiment (Experiment 2; audio-visual regular primes: RegAV, auditory-only irregular primes: IrregA) compared to the experiment from Canette et al. (2019), *n* = 25 (auditory-only regular primes: RegA, auditory-only irregular primes: IrregA). Regular and irregular rhythms were identical in both experiments; however, in Experiment 2, a bouncing figure was added to the regular rhythms, and a static image of this same figure was added to the irregular rhythms. Boxplots represent the distribution of data as implemented in ggplot2 in R (R Core Team, 2018), with the black line representing the median, the box representing the interquartile range, the whiskers presenting the spread of data, and extra points representing potential outliers. Individual lines represent individual participant data, and the asterix represents the mean.

The paired-samples *t*-test showed that there was no significant difference between performance after RegAV primes (*M* = 1.92, *SD* = 1.07) compared to IrregA primes (*M* = 2.07, *SD* = 0.84), *t*(30) = 0.73, *p* = 0.47. This result was surprising, as irregular rhythms typically result in poorer performance on grammaticality judgments compared to regular rhythms (Przybylski et al., 2013; Bedoin et al., 2016; Chern et al., 2018; Canette et al., 2019, 2020a; Fiveash et al., 2020; Ladányi et al., 2021). To investigate whether the addition of a bouncing figure was detrimental to subsequent performance, we compared the present results to the results of Canette et al. (2019), see Figure 4. The between-subjects analysis showed that performance after the RegAV condition of the present experiment was reduced compared to performance in the RegA condition of Canette et al. (2019), *t*(54) = 1.96, *p* = 0.054, *d* = 0.53, even though this difference just fell short of significance. There was no difference in performance after irregular primes in the present experiment (IrregA) compared to after irregular primes (IrregA) in Canette et al. (2019), *t*(54) = 0.06, *p* = 0.96. The comparison of these two datasets suggests that the addition of an audio-visual bouncing figure removed the benefit of the regular rhythmic primes.

#### Response Bias c

The paired-samples *t*-test showed no difference between the two conditions for response bias *c*, *t*(30) = 0.26, *p* = 0.80, as both RegAV (*M* = 0.53, *SD* = 0.49), *t*(30) = 6.05, *p* < 0.001, *d* = 1.09 and IrregA (*M* = 0.51, *SD* = 0.44), *t*(30) = 6.50, *p* < 0.001, *d* = 1.17 showed a significant bias to respond grammatical. Between-subjects, there were no differences in response bias for RegAV compared to RegA, *t*(54) = 0.05, *p* = 0.96 in Canette et al. (2019), or between the two irregular auditory conditions, *t*(54) = 0.54, *p* = 0.59.

#### Barcelona Musical Reward Questionnaire

The multiple linear regression model showed that the difference score of RegAV minus IrregA was not predicted by any of the sub-scales of the BMRQ (all *p*-values > 0.10), and that the model was not significant, *F*(5,25) = 1.96, *p* = 0.12, with an *r*^2^ of 0.28 (adjusted *r*^2^ = 0.14). However, the backward stepwise model showed that the sensory-motor sub-scale was a significant negative predictor of the difference score (Estimate = −0.03, *t* = −2.08, *p* = 0.047), and the model including the sensory-motor sub-scale and the mood regulation sub-scale (Estimate = −0.03, *t* = −1.39, *p* = 0.17) was significant, *F*(2,28) = 4.77, *p* = 0.02, *r*^2^ = 0.25, adjusted *r*^2^ = 0.20. The negative contribution of the sensory-motor scale (i.e., how strongly does music induce body movements within individuals) suggests that participants’ sensory-motor sensitivity may predict how they are affected by the bouncing figure. Those with *low* sensory-motor scores performed *better* after the bouncing figure, whereas those with *high* sensory-motor scores did *worse* after the bouncing figure. It is possible that participants with low scores were aided by the bouncing figure to extract the beat, whereas those who already had high sensory-motor sensitivity were more distracted by the figure, or aligned themselves to different aspects of the bouncing figure (e.g., the hands, which were not necessarily fully aligned with the beat).

The results of Experiment 2 provide further evidence for a detrimental effect of the point-light figures on grammaticality judgments. However, considering that the bouncing movement was aligned to the knee flexion, and the other body parts were not controlled (i.e., they were naturally moving so could have created antiphase or unsynchronized movements), we decided to change our point-light figure movement to a swaying figure, with clear and precise hip movements aligned with the beat. The swaying movement also allowed the figure to be forward-facing, which may have been important to the perception of the figure as a person.

## Experiment 3

### Participants

Thirty-one native French speaking adults participated in Experiment 3 (*M*_*age*_ = 20 years, *SD* = 1.9; range = 18–26; 26 women) and were recruited from Universities in Lyon and social media. On average, participants had 3.61 years (*SD* = 4.24; range = 0–13) of musical experience (including years of classes and years of individual playing). Nineteen participants reported that they had previously played music (*M* = 5.89 years, *SD* = 3.97, range = 1–13), and eight reported to currently play music. Seventeen participants reported attending dance classes in the past, and two currently attended dance classes. Participants reported no history of dyslexia or neurological issues, and no issues with hearing or vision that precluded them from participating in the study. All participants provided written informed consent, as approved by the French ethical committee (*Comité de Protection des Personnes Ile de France X, CPP*). They were paid 12 euros an hour for their participation.

### Results and Discussion

#### D Prime

**FIGURE 5:**
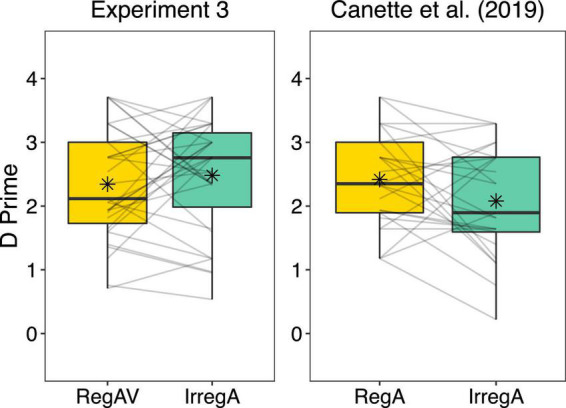
Comparison of the current audio-visual (AV) experiment (Experiment 3; audio-visual regular primes: RegAV, auditory-only irregular primes: IrregA) compared to the experiment from Canette et al. (2019), *n* = 25 (auditory-only regular primes: RegA, auditory-only irregular primes: IrregA). Regular and irregular rhythms were identical in both experiments; however, in Experiment 3, a swaying figure was added to the regular rhythms, and a static image of this same figure was added to the irregular rhythms. Boxplots represent the distribution of data as implemented in ggplot2 in R (R Core Team, 2018), with the black line representing the median, the box representing the interquartile range, the whiskers presenting the spread of data, and extra points representing potential outliers. Individual lines represent individual participant data, and the asterix represents the mean.

The paired-samples *t*-test showed no significant difference in performance after RegAV primes (*M* = 2.34, *SD* = 0.88) compared to IrregA primes (*M* = 2.48, *SD* = 0.90), *t*(30) = 0.82, *p* = 0.42 in the current experiment. Between-subjects, there was no significant difference in performance after RegAV primes in the current experiment compared to the RegA primes in Canette et al. (2019), *t*(54) = 0.33, *p* = 0.74. However, compared to the IrregA condition in Canette et al. (2019), performance after the IrregA condition in the current audio-visual experiment appeared somewhat higher, though not significantly, *t*(54) = 1.69, *p* = 0.10 (Figure 5).

#### Response Bias c

There was no significant difference between conditions, *t*(30) = 0.64, *p* = 0.53. Participants were significantly biased to respond grammatical in both the RegAV (*M* = 0.54, *SD* = 0.46), *t*(30) = 6.52, *p* < 0.001, *d* = 1.17 and the IrregA (*M* = 0.61, *SD* = 0.32), *t*(30) = 10.71, *p* < 0.001, *d* = 1.92, conditions. There were also no significant differences in response bias for RegAV compared to RegA, *t*(54) = 0.05, *p* = 0.96 in Canette et al. (2019), or between the two irregular auditory conditions, *t*(54) = 1.61, *p* = 0.11.

#### Barcelona Musical Reward Questionnaire

The multiple linear regression model showed that the difference score of RegAV minus IrregA was negatively predicted by the sensory-motor sub-scale (Estimate = −0.06, *t* = −2.78, *p* = 0.01). The social reward sub-scale was approaching significance (Estimate = 0.03, *t* = 1.73, *p* = 0.096), and no other sub-scales were significant (all *p*-values > 0.46). However, the model itself did not reach significance, *F*(5,25) = 2.18, *p* = 0.09, with an *r*^2^ of 0.30 (adjusted *r*^2^ = 0.16). The backward stepwise model with two sub-scales was significant, *F*(2,28) = 4.67, *p* = 0.02, *r*^2^ = 0.25, adjusted *r*^2^ = 0.20: it confirmed that the sensory-motor sub-scale was a significant negative predictor of the difference score (Estimate = −0.05, *t* = −2.59, *p* = 0.01), and that the social reward sub-scale was a positive predictor of the difference (Estimate = 0.04, *t* = 2.63, *p* = 0.01). Taken together, these results support the results from Experiment 2 (bouncing figure), that participants with low sensory-motor sensitivity performed better after watching the swaying figures, but that participants with high sensory-motor sensitivity performed worse after watching these figures. Additionally, the positive social reward sub-scale predictor suggests that participants who scored *higher* on the social reward sub-scale (i.e., related to the social bonding aspect of music) also performed *better* after the audio-visual rhythms. The swaying figure was facing directly toward the participants, compared to the bouncing figure, which was facing to the side. It is possible that watching another “person” swaying in time to the rhythms may have aided the participants who had high social reward from music in extracting the beat.

## General Discussion

Across three experiments in children (Experiment 1) and adults (Experiments 2 and 3) we found that adding a visual cue in the form of a point-light figure bouncing (Experiments 1 and 2) or swaying (Experiment 3) to a regular rhythmic prime did not enhance subsequent grammaticality judgments of naturally spoken sentences. There was evidence to suggest that the addition of this visual figure instead removed the typically observed benefit of the rhythmic prime, and was detrimental to beat-based perception. Interestingly, individual differences appeared to affect how the visual cue influenced the participants. As RA in children increased, they performed better after both the regular audio-visual primes and the regular auditory primes compared to children with lower RAs; however, this effect remained significant after multiple comparison correction only for the grammaticality judgments after RegAV primes. In both Experiments 2 and 3, adults who scored lower on the sensory-motor subscale of the BMRQ appeared to be aided by the visual cue (better performance after RegAV compared to IrregA primes), whereas those who scored higher on this scale performed worse after the RegAV primes. Additionally, for the swaying figure of Experiment 3, participants with higher social scores on the BMRQ performed better after the RegAV prime compared to the IrregA prime.

### Detrimental Effect of Visual Cue on Grammaticality Judgments

The current results suggest that adding a visual cue to a regular rhythmic prime reduces the beneficial effect of the prime compared to regular primes presented only auditorily. In Experiment 1, children performed significantly worse on the grammaticality task after a RegAV prime compared to a RegA prime, directly showing that the addition of a point-light bouncing figure reduced the effect of the prime on performance. In Experiments 2 and 3, adults were presented with RegAV and IrregA primes. No differences were found between these two conditions in either experiment. However, considering the typical finding of a beneficial effect of the regular compared to irregular primes, we compared these results to the pure auditory experiment of Canette et al. (2019). In Experiment 2, between-subjects analyses showed that performance in the RegAV condition was reduced in comparison to the RegA data in Canette et al. (2019), while the IrregA condition was comparable to performance with the same primes in Canette et al. (2019). These results suggest that for adults, the addition of the visual cue reduced the beneficial effect of the regular prime. The between-subjects comparison was inconclusive for Experiment 3, with no difference between RegAV and RegA or between IrregA and IrregA when compared to Canette et al. (2019).

Together with previous findings, the current results suggest that passively watching a visual point-light figure does not necessarily enhance rhythm and beat processing. Previous research involving a cueing component showed that including auditory-motor manipulations seem to enhance the processing of the rhythm (e.g., Cason et al., 2015; Falk and Dalla Bella, 2016; Falk et al., 2017). However, visual cues without additional movement from the participant have shown mixed effects on rhythm processing (positive: Su, 2014b; no effects: Phillips-Silver and Trainor, 2005, 2007; Su, 2014a), and the positive effects were shown for a same-different task and a synchronization task with small sample sizes (14 and 11 plus author, respectively; Su, 2014b). To our knowledge, the current experiments were the first to add a moving point-light figure to a rhythmic priming experiment. Our aim was to use the moving point-light figures to enhance the activation of the auditory-motor connection in the brain (Saygin et al., 2004; Saygin, 2007), thereby improving rhythmic entrainment and enhancing subsequent sentence processing. Instead, the visual figure appeared to remove the benefit of the regular rhythm, suggesting weaker entrainment to the rhythm and/or disturbing effects due to the additional visual information.

The addition of the visual point-light animation may have reduced or interrupted rhythmic entrainment. For example, the extra visual information may have been distracting to participants, especially if it was perceived as an additional source of beat-based information that was not integrated with the rhythm. Su (2014a) discussed the potential extra demands of adding a visual cue on working memory capacity, which may have removed potential beneficial effects of an extra cue to processing the beat. It is possible that the visual cue and auditory information were not integrated in the current experiment, even though the visual figures were synchronizing to the rhythm of the musical prime, with the goal to encourage an integrated audio-visual percept.

The potential effect of a visual cue might also depend on the task involved and the implementation of the cue, and it is possible that the rhythmic priming paradigm does not benefit from a visual cue under the current circumstances. The successful use of a moving point-light figure in Su (2014b) included a same-different task and a synchronization task, whereby the point-light figure continued to bounce during the tasks (e.g., in the final rhythm for the same-different task and during the synchronization task). In contrast, a rhythm reproduction task (with an accompanying visual figure during perception, but no accompanying visual figure during the reproduction phase) and a same-different task on weakly metrical rhythms did not show an effect of the visual cue (Su, 2014a). Watching a visual cue for 2 min without moving along also did not influence recognition of test sequences in the trained meter, suggesting that for longer stimuli, visual cues alone do not influence beat perception (Phillips-Silver and Trainor, 2005, 2007). Further, even with auditory-motor training (without a visual cue), Cason et al. (2015) did not show an improvement after the matching cue, but rather reduced performance on *mismatching* cues. It therefore appears that the effectiveness of a visual cue (here, a point-light figure) depends strongly on the type of stimuli, the task, and possibly the stimulus duration (i.e., perhaps participants paid less attention to the cue over time). The current rhythms were complex in the sense that they contained multiple instruments; however, the beat was clear and isochronous throughout a long timeframe whereby the same cycle was repeated for 32 s, making it relatively easy to extract the beat. It is therefore interesting to investigate individual differences that might be expected to relate to success in beat-based processing.

### Individual Differences

Across the three experiments, individual differences appeared to influence how the audio-visual figure affected performance on grammaticality judgments. For children, the correlation between RA and RegAV remained significant after correction for multiple comparisons, while the correlation between RA and RegA fell short of significance, suggesting that the connection between RA and benefit of the visual cue may have been slightly stronger for children with higher RAs. The correlation of RA (and not CA) with performance after regular rhythmic primes was also observed in Fiveash et al. (2020). Links between language skills (e.g., grammar, phonological awareness, reading) and rhythm processing have been shown in the literature (Tierney and Kraus, 2013; Gordon et al., 2015a,b) and research is suggesting a link between speech and language impairments and rhythm processing (Ladányi et al., 2020; Fiveash et al., 2021). It is therefore possible that children with higher RAs were better able to use the visual cue to scaffold their beat perception and enhance its effect on subsequent speech processing.

The adult experiments showed that the effect of the visual cue differed depending on participants’ scores on the sensory-motor and social sub-scales of the BMRQ (Mas-Herrero et al., 2013). The sensory-motor sub-scale included the questions: (1) *I don’t like to dance, not even with music I like (reverse scored)*, (2) *music often makes me dance*, (3) *I can’t help humming or singing along to music that I like, and* (4) *when I hear a tune I like a lot I can’t help tapping or moving to its beat.* In both adult experiments, participants who scored *lower* on the sensory-motor scale performed *better* after the RegAV rhythmic primes than the IrregA primes, whereas participants who scored *higher* on this scale performed *worse* after the RegAV primes than the IrregA primes. Previous research has suggested that participants use a visual cue more strongly when it is difficult to extract a beat from the auditory information (Su, 2014b). The authors interpret this finding within the *principle of inverse effectiveness* of multisensory integration, which suggests that as sensitivity to a unimodal stimulus decreases, the value of the multimodal cue increases (Meredith and Stein, 1986; Senkowski et al., 2011). In the current experiment the difficulty of the auditory rhythms was not manipulated. However, previous research has suggested that individual performance on a task can evoke the principle of inverse effectiveness (Caclin et al., 2011; Albouy et al., 2015). It is therefore possible that participants who were poorer at sensory-motor integration found it more difficult to extract a beat, and therefore relied more on the visual cue. Conversely, participants who had no trouble extracting the beat from the rhythms might have been more distracted by the bouncing figure, or focused on other body parts that were not necessarily aligned. Although with the current dataset it is not possible to investigate the level of attention or distraction during prime presentation, the current results suggest that individual differences should be monitored in future audio-visual experiments, and a measure of attention should be introduced.

In addition to the sensory-motor sub-scale influence, the social sub-scale of the BMRQ was a positive predictor of the difference between RegAV and IrregA performance in Experiment 3. This finding suggests that as participants scored higher on the social sub-scale, their performance was better after RegAV primes than IrregA primes, and as they scored lower on the social sub-scale, their performance was worse after RegAV primes. The social sub-scale included the questions: (1) *when I share music with someone I feel a special connection with that person*, (2) *music makes me bond with other people*, (3) *I like to sing or play an instrument with other people*, and (4) *at a concert I feel connected to the performers and the audience.* It is possible that participants with higher social scores were more engaged with the swaying figure on the screen, and therefore paid more attention to the visual cue, enhancing beat perception.

This suggestion is supported by research suggesting that in typically developing populations: (a) participants with higher empathy looked longer at social images than participants with lower empathy, who were quicker and more frequent to look away from social images (Hedger et al., 2018), (b) participants with fewer autistic traits became more precise over an experimental session for social, but not non-social stimuli compared to participants with higher autistic traits (Honisch et al., 2021), and (c) participant pairs with high empathic perspective taking were better at synchronizing together than participant pairs with low empathic perspective taking (Novembre et al., 2019). Taken together with the current results, it is possible that participants with higher social scores may have been more interested and paid more attention to the swaying visual cue, possible resulting in enhanced auditory-motor entrainment, resulting in a benefit of the visual cue. This interpretation is somewhat supported by the observation that the same pattern of results regarding the social scale was not observed for the bouncing figure in Experiment 2. It is indeed possible that the forward-facing swaying figure was more appealing and looked more “social” than the bouncing figure, which was facing to the left (to show more clearly the knee bend).

### Limitations and Future Directions

There were some important differences in our study compared to previous studies showing enhanced beat-based processing with motor movement (e.g., Phillips-Silver and Trainor, 2007; Falk et al., 2017) or with a visual point-light figure (Su, 2014b). It might be that the motor movement element could be critical to enhance the auditory-motor link and its potential benefit for beat processing. We gave free instruction to our participants and told them that they were allowed to move along with the bouncing/swaying figure if they wanted to. However, we did not enforce the movement of participants, and other than observational data from the children, we did not record whether participants did move along with the rhythms (and if they did, whether they moved in time). Future research could more clearly activate this motor link and ask participants to directly move or tap in time with the visual figure. If a motor component is implemented, it would be important to monitor accuracy, as unsynchronized motor movements could be detrimental to beat-based processing. Further, a similar motor component should be introduced for the irregular rhythms to control for attention and task engagement; however, this implementation could be more difficult to define for irregular rhythms. The present experiments included a small training session so that participants were trained to focus on the synchronization of the point-light figure with the music, but a longer familiarization or training session (i.e., rhythmic tapping or rhythm workshop beforehand, as in Hidalgo et al., 2017) might have been necessary to enhance the effect of the visual cue.

Based on studies suggesting that moving visual cues are effective for synchronization (Silva and Castro, 2016; Torres et al., 2019), the inclusion of such a cue could particularly benefit beat perception and synchronization to rhythmic primes when a motor component is involved. This hypothesis is in line with auditory-motor studies showing enhanced cueing effects with additional motor synchronization (Falk and Dalla Bella, 2016; Falk et al., 2017). Therefore, we would predict that asking participants to synchronize with the regular primes might provide an enhanced rhythmic priming effect compared to purely auditory primes. However, it is also possible that adding an additional tapping task while also watching a visual moving figure and listening to the rhythms might become too complex if the full audio-visual percept cannot be integrated and/or the required additional tapping task taxes perception and cognitive resources. Future research could test these effects and investigate whether and how visual cues and the addition of movement could be used effectively to enhance synchronization within rhythmic priming paradigms. Individual differences would also be important to measure, as participants would most likely vary in their synchronization abilities, which could affect the quality of synchronization (e.g., Doelling and Poeppel, 2015; Assaneo et al., 2019).

Together with previous results, it appears that visual cues may be beneficial only under certain circumstances, in particular relating to attention and precision of the visual cue. Attending to the stimulus for 32 s may have been too long to maintain attention with the visual cue. In Su (2014b), attention was maintained (over short auditory sequences) by asking participants to additionally detect if one of the points on the point-light figure changed color. In future experiments investigating the influence of visual cues on rhythmic priming, it would be valuable to add an attentional check to measure attention to the cue and how it fluctuates over time. The stimuli in Su (2014b) were also carefully controlled. Recordings of natural movement were made and then manipulated to be more precise. Movement along the horizontal axis was removed, the feet markers were kept still even though they naturally moved in the original recording, and the least temporal deviation of one cycle of movement was used across all rhythms. In contrast, we had a musician bouncing or swaying in time with the rhythms for 32 s and did not manipulate this movement artificially. Therefore, our stimuli could be considered natural, but there were also likely to be small timing and movement deviations, which could have influenced the precise synchronization of the visual cue with the music. For example, movements of other body parts (e.g., arms, hands, and head) may not have been synchronized with the beat, and participants may have focused on these body parts rather than the aligned knee movements of the bouncing movement. For this reason, we changed our stimuli in Experiment 3 to a swaying figure with the aim to have more precise and clear visual cues to the beat, even though there was still free movement of the other body parts (e.g., arms, hands, and head). However, even with this manipulation there was still no advantage for the visual cue. The addition of a visual cue may therefore aid rhythm perception in particular for short rhythmic sequences and with very precise timing.

Finally, our findings revealed the potential influence of individual differences in whether the visual cue enhanced rhythmic entrainment or not. Future research could consider testing a larger sample of participants and including objective measures such as rhythm perception skills, as well as empathy and other social traits, for example. Note that the successful audio-visual experiments in Su (2014b) contained small sample sizes, with 14 participants in the same-different task and 11 participants (plus the author) in the synchronization task. It is possible that the included participants in Su’s study were more strongly influenced than the general population by the visual cue (especially the author, who would have been very familiar with the visual cue). Future research investigating the influence of visual cues could therefore include manipulations of task, stimulus duration, and individual differences, as these appear to play important roles in the efficacy of the visual cue.

## Conclusion

The current study showed that passively watching a visual point-light figure moving in time to regular rhythms removed the benefit of regular rhythmic primes on subsequent grammaticality processing. These results suggest that in the current experimental paradigm, the addition of a visual cue does not enhance beat perception and may detract from rhythmic entrainment. However, individual differences appeared to play an important role, as participants with low sensory-motor sensitivity benefited more from the visual cue, as did participants with high social sensitivity when the visual cue was facing forwards rather than sideways. The current study suggests that the task, the visual cue implementation, attention, and individual differences are all important elements as to whether visual cues aid beat perception and potentially benefit the rhythmic priming effect.

## Data Availability Statement

The raw data supporting the conclusions of this article will be made available by the authors, without undue reservation.

## Ethics Statement

The studies involving human participants were reviewed and approved by Comité de Protection des Personnes (CPP), France. For Experiment 1 (involving children), written informed consent to participate in this study was provided by the participants’ legal guardian/next of kin. For Experiments 2 and 3 (involving adults), participants provided their written informed consent to participate in the study.

## Author Contributions

AF, L-HC, NB, and BT designed the study. AF and L-HC analyzed the data. AF wrote the first draft of the manuscript. AF, L-HC, and NB tested participants. AF, BB, L-HC, and BT contributed to the design of the point-light figures. BB provided the point-light figures. AF and BT completed and finalized the analyses and result presentation. BT and NB provided funding and resources. All authors edited the manuscript and interpreted the results.

## Conflict of Interest

The authors declare that the research was conducted in the absence of any commercial or financial relationships that could be construed as a potential conflict of interest.

## Publisher’s Note

All claims expressed in this article are solely those of the authors and do not necessarily represent those of their affiliated organizations, or those of the publisher, the editors and the reviewers. Any product that may be evaluated in this article, or claim that may be made by its manufacturer, is not guaranteed or endorsed by the publisher.

## Footnotes

1 A 2 × 2 mixed ANOVA was not run because there was no IrregAV condition. Therefore, we directly tested the contrast of interest (RegAV vs. RegA), and we additionally tested whether there was a difference in “baseline” (auditory only) irregular performance (IrregA vs. IrregA) between the two experiments.

2 Though note that a Fisher *z* test showed no significant difference between the correlations, *z* = 0.44, *p* = 0.33.
