# Supplementary material for: When Visual Cues Do Not Help the Beat: Evidence for a Detrimental Effect of Moving Point-Light Figures on Rhythmic Priming
Source: Front Psychol. 2022 Feb 4;13:807987. doi: 10.3389/fpsyg.2022.807987 (PMC8855071; doi:10.3389/fpsyg.2022.807987)
Supplement: Supplementary file 1 [file Table_1.DOCX]

| **Supplementary Table 1**  *Example Grammatical Errors* | | |
| --- | --- | --- |
| Experiment 1: Child Sentences | | |
| Error Type | Sentence | Explanation |
| Number Agreement | L’air est (**sont**) pur dans la montagne  *The air is (****are****) pure in the mountain* | The third person plural (sont) is used instead of the third person singular (est) |
| Person Agreement | Les enfants jouent (**jouons**) dans le jardin  *The children are playing (*********) in the garden* | The first person plural (jouons) is used instead of the third person plural (jouent) |
| Gender Agreement | Le vent souffle sur la (**le**) colline ce soir  *The wind is blowing on the_FEM_ (the_MASC_) hill tonight* | “The hill” is feminine, but a masculine determiner is given |
| Tense Agreement | Il est possible qu'Antoine soit (**est**) en vacances cette semaine  *It is possible that Antoine is (*) on vacation this week* | Indicative present (est) is used instead of subjunctive present (soit) |
| Auxiliary | Aujourd'hui, Marie est (**a**) rentrée tôt chez elle  *Today, Marie went (*) home early* | The verb rentrer must be conjugated with the auxiliary “être” (to be), and not the auxiliary “avoir” (to have). |
| Morphology | Je rentre chez moi en (**par**) voiture tous les soirs  *I drive home by (*********) car every night* | Wrong proposition |
| Position | Il travaille avec moi, je le connais (**le**) bien depuis longtemps  *He works with me, I have (****him****) known him well for a long time* | The word “le” should be before the verb “connais”, not after it. |
| Past Participle | Les enfants ont vu (**voir**) un grand chien  *The children saw (****have see****) a big dog* | The past tense conjugation of the past participle of the verb was not made. |
| Experiment 2: Adult Sentences | | |
| Error Type | Sentence | Explanation |
| Tense Agreement | J’espère que vous avez (**ayez**) pris la meilleure decision  *I hope that you have (*) made the best decision* | Subjunctive present (ayez) is used instead of the indicative present (avez) |
| Preposition | Il a demandé à (**de**) voir la nouvelle classe  *He asked to (*********) see the new class* | The verb “demander” needs “à” rather than “de” in this situation |
| Person Agreement | C’est moi qui ai (**a**) oublié les clefs sur la porte  *I’m the one who (*) left the keys in the door* | Third or second person singular (a, as) is used instead of first person singular (ai) |

Notes: Ungrammatical version presented in bold and brackets. English translation in italics. Note that the lists were created so that participants only heard one version of each sentence (grammatical or ungrammatical). See all matched lists in Fiveash et al. (2020) for child sentences and Canette et al. (2019) for adult sentences.

*There is no direct translation for this error in English.
